# An Injectable Living Hydrogel with Embedded Probiotics as a Novel Strategy for Combating Multifaceted Pathogen Wound Infections

**DOI:** 10.1002/adhm.202400921

**Published:** 2024-07-14

**Authors:** Siyuan Tao, Sixuan Zhang, Kongchang Wei, Katharina Maniura‐Weber, Zhihao Li, Qun Ren

**Affiliations:** ^1^ Laboratory for Biointerfaces Empa Swiss Federal Laboratories for Materials Science and Technology St. Gallen 9014 Switzerland; ^2^ Laboratory for Biomimetic Membranes and Textiles Empa Swiss Federal Laboratories for Materials Science and Technology St. Gallen CH 9014 Switzerland

**Keywords:** biofilm, injectable hydrogel, living material, probiotics, wound dressing

## Abstract

Wound infections pose a significant challenge in healthcare, and traditional antibiotic treatments often result in the development of resistant pathogens. Addressing this gap, ProGel is introduced, a living hydrogel created by entrapping probiotic *Lactobacillus plantarum* as a therapeutic component within a gelatin matrix. With a double‐syringe system, ProGel can be easily mixed and applied, conforming swiftly to any wound shape and forming hydrogel in situ. It also demonstrates robust mechanical and self‐healing properties owing to the Schiff‐base bonds. ProGel sustains more than 80% viability of the entrapped *L. plantarum* while restricting their escape from the hydrogel. After a week of storage, more than 70% viability of the entrapped *L. plantarum* is preserved. Importantly, ProGel exhibits broad‐spectrum antimicrobial efficacy against pathogens commonly associated with wound infections, i.e., *Pseudomonas aeruginosa* (7Log reduction), *Staphylococcus aureus* (3‐7Log reduction), and *Candida albicans* (40–70% reduction). Moreover, its cytocompatibility is affirmed through coculture with human dermal fibroblasts. The effectiveness of ProGel is further highlighted in more clinically relevant tests on human skin wound models infected with *P. aeruginosa* and *S. aureus*, where it successfully prevents the biofilm formation of these pathogens. This study showcases an injectable living hydrogel system for the management of complex wound infections.

## Graphical abstract

ProGel, an innovative living hydrogel incorporating *Lactobacillus plantarum*, addresses wound infections by offering broad‐spectrum antimicrobial action against pathogens like *Pseudomonas aeruginosa* and *Staphylococcus aureus*. Applied via a double‐syringe system, ProGel adapts to any wound shape, demonstrating robust mechanical properties and sustained probiotic viability. This system represents a promising advance in managing complex wound infections.

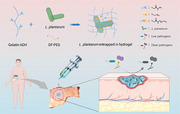

## 1 Introduction

Wound infections pose a significant crisis in healthcare, leading to prolonged treatment and healing times, increased morbidity, and substantial healthcare costs.^[^ ^1^ ^]^ At the same time, antibiotic resistance accounts for ≈33 000 deaths annually in the EU, and it has been associated with an estimated €1.5 billion in healthcare costs and productivity loss.^[^ ^2^ ^]^ Among the pathogens commonly associated with wound infections, *P. aeruginosa*, *S. aureus*, and *C. albicans* stand out as major culprits, known for their virulence and resistance to conventional antibiotics.^[^ ^3^ ^]^ These pathogens can form biofilms that impede the normal wound‐healing process, leading to prolonged inflammation and delayed healing. Moreover, these biofilms provide inherent resistance to antimicrobial agents, including antibiotics, making it challenging to eliminate these pathogens and increasing the risk of chronic infections.^[^ ^3^, ^4^ ^]^

As a response to the above‐mentioned challenges and especially addressing antibiotic resistance, various alternative methods have been explored to treat wound infections, including antimicrobial peptides (AMPs),^[^ ^5^ ^]^ antimicrobial photodynamic therapy (aPDT),^[^ ^6^ ^]^ silver‐based dressings,^[^ ^7^ ^]^ etc. While all of these methods have shown potential as alternatives to antibiotics, probiotics stand out as an intriguing approach due to their unique mechanism of action and potential benefits.^[^ ^8^ ^]^ These beneficial bacteria have been extensively studied and utilized in various fields of medicine, including gastrointestinal health, immune modulation, and more recently, wound management. Probiotics can inhibit the growth of pathogens through competition for nutrients and adhesion sites, modulation of the immune response, and production of antagonistic substances such as lactic acid, enzymes, exopolysaccharides (EPS), etc.^[^ ^8^ ^]^ *L. plantarum*, specifically, has shown significant antimicrobial activity against a wide range of wound pathogens, including *P. aeruginosa*, *S. aureus*, and *C. albicans*.^[^ ^9^ ^]^ Numerous in vitro and in vivo studies have indicated that the direct topical administration of *L. plantarum* accelerates wound healing by suppressing pathogen infections, modulating inflammatory reactions, and improving tissue repair.^[^ ^10^ ^]^ However, directly applying probiotics to wounds may introduce complications.^[^ ^11^ ^]^ Their tendency for quick growth and colonization is linked to the risk of uncontrolled spread. When they encounter the immune system directly, their effectiveness could get significantly reduced.^[^ ^12^ ^]^ Additionally, the potential displacement of probiotics from the infection site may require repeated applications.^[^ ^11^, ^12^ ^]^ Other difficult conditions in wounds, such as varying pH levels and antibiotics, may also weaken the effectiveness of probiotics.^[^ ^11^ ^]^ Hence, designing a carrier system that controls the release of probiotics into wounds is crucial. This system should leverage the bioactive substances secreted by probiotics to treat infections while preventing the bacteria from spreading into the surrounding area and causing serious infections.

**Figure 1:**
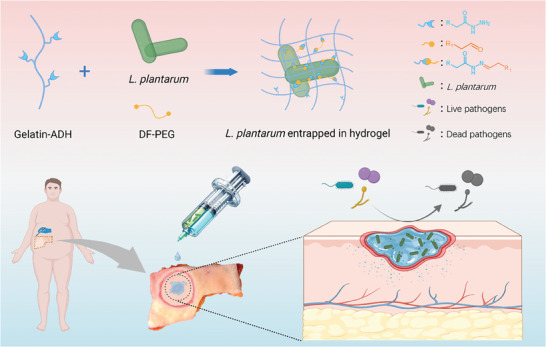
Schematic illustration of Probiotic‐Entrapping ProGel Hydrogel for Wound Treatment. This schematic highlights ProGel, a living hydrogel formed by incorporating *L. plantarum* into a matrix of adipic acid dihydrazide (ADH)‐modified gelatin crosslinked with benzaldehyde‐functionalized polyethylene glycol (DF‐PEG). The double‐syringe system facilitates the precise mixing and application of ProGel, allowing it to adapt seamlessly to the contours of different wounds. ProGel's unique design permits the diffusion of probiotic‐secreted antibacterial agents to the wound site, actively inhibiting the growth of prevalent wound pathogens. The figure underscores the innovative potential of this injectable living hydrogel system in advancing treatment strategies for complex wound infections.

Hydrogels as versatile biomaterials are well‐suited for wound management due to their unique properties. They act as a protective barrier, managing exudate and shielding the wound from external contaminants as well as mechanical trauma. Living hydrogels, incorporating probiotics within hydrogels for wound treatment present distinct advantages over direct administration. The cross‐linked network can provide regulated drug release, ensuring their sustained presence in the wound for enhanced therapeutic efficacy.^[^ ^13^ ^]^ The hydrogel matrix also acts as a protective barrier for the probiotics against adverse wound environments, and its inherent biocompatibility reduces inflammatory responses.^[^ ^14^ ^]^ Moreover, its porous architecture promotes requisite nutrient exchange, bolstering probiotic viability.^[^ ^11^, ^12^, ^15^ ^]^ Among the various hydrogel systems, Schiff‐base hydrogels exhibit exceptional promise to entrap and regulate the release of therapeutic agents thanks to their reversible dynamic covalent bonds formed during cross‐linking.^[^ ^15c^ ^]^ These bonds endow the hydrogels with enhanced mechanical stability, multiresponsiveness, and self‐healing properties. While the multiresponsiveness allows the embedded therapeutic components to release in a “smart” manner, the self‐healing property enables the hydrogel to withstand mechanical stresses and maintain its structural integrity in dynamic wound environments, thereby minimizing potential side effects of burst release caused by unexpected mechanical ruptures.^[^ ^16^ ^]^ Gelatin, a collagen derivative, is favored due to its biocompatibility, biodegradability, and ease of application.^[^ ^17^ ^]^ It has been widely studied as a beneficial scaffold for enhancing cell migration and wound healing.^[^ ^18^ ^]^ Its amine groups can engage with aldehyde groups in alkaline conditions to establish Schiff‐base bonds. Furthermore, carboxyl groups on gelatin, when functionalized with acylhydrazide groups using carbodiimide chemistry, allow Schiff‐base reactions at neutral pH, and augment bond robustness. This crosslinking method avoids the need for potentially detrimental UV initiation, preserving the integrity and viability of the entrapped probiotics.^[^ ^19^ ^]^

In this study, we detail a refined gelatin‐based hydrogel system crosslinked via Schiff‐base chemistry, optimized for probiotic immobilization to tackle complex wound infections (**Figure** **1**). This is the first application of a gelatin‐based Schiff‐base hydrogel as a carrier for probiotics in wound management. Employing a dual‐syringe mechanism, the gelatin‐based hydrogel can form in situ and seamlessly adapt to complex wound geometries for better therapeutic outcomes. *L. plantarum* was successfully entrapped in the hydrogel and compatibility with, as well as sustained retention of the probiotics was demonstrated. This design effectively minimizes probiotic dispersal, maintaining their concentrated presence at the wound interface. Remarkably, these entrapped probiotics exhibit potent antimicrobial activity against a broad pathogenic array. Our approach offers a multitargeted action, combating gram‐negative (*P. aeruginosa*), gram‐positive (*S. aureus*), and fungal (*C. albicans*) pathogens concurrently. Assessments using an ex vivo human skin model confirmed the viability of the encapsulated probiotics in physiological conditions and the strong antimicrobial properties of our probiotic‐laden hydrogel system.

## 2 Results and Discussion

### 2.1 Synthesis of hydrazide‐functionalized gelatin (Gel‐ADH) and difunctional PEG (DF‐PEG)

To fabricate hydrogel with improved material properties for encapsulation of probiotics, gelatin and PEG were utilized and chemically modified.

**Figure 2:**
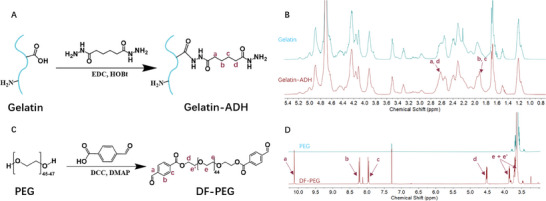
Chemical Modification and Confirmation of Gelatin and PEG. A) Gel‐ADH was synthesized by reacting gelatin's carboxyl groups with ADH. B) The successful modification of gelatin is evident in the 1H NMR spectra, with signal peaks at δ 1.9 and 2.6 ppm. C) Hydroxyl‐terminated PEG was esterified with FA to form DF‐PEG. D) The synthesized DF‐PEG, displaying characteristic 1H NMR peaks at 10.1 and 7.9–8.3 ppm, attributed to the aldehyde and aromatic protons, respectively, confirms the successful synthesis.

The Gel‐ADH was synthesized by employing carbodiimide chemistry, whereby the carboxyl groups of gelatin reacted with the hydrazide groups of adipic acid dihydrazide (ADH) (**Figure** **2**A). The successful modification was evident in the ^1^H NMR spectra (Figure 2B). The presence of methylene signal peaks at δ 1.9 and 2.6 ppm confirmed that gelatin underwent successful modification with ADH. The extent of hydrazide modification, crucial for the gelation process, was assessed by quantifying the reaction of trinitrobenzene sulfonic acid (TNBS) with the primary amino groups in the gelatin before and after the chemical modification. The functionalization degree was found to be 36%, which aligns with the result of a previous study.^[^ ^20^ ^]^

In parallel, the DF‐PEG was synthesized by esterifying hydroxyl‐terminated PEG with 4‐formylbenzoic acid (FA) (Figure 2C). This modification strategy was chosen to form dialdehyde derivatives of PEG, with greater stability and lesser toxicity compared to small molecular dialdehydes such as glyoxal and glutaraldehyde, which have been demonstrated to exhibit mutagenic and dermal sensitization effects,^[^ ^21^ ^]^ respectively. The synthesized DF‐PEG displayed characteristic ^1^HNMR peaks for PEG and FA, affirming successful synthesis (Figure 2D). The peaks at 10.1 and 7.9–8.3 ppm were attributed to the aldehyde and aromatic protons, respectively. By calculating the ratio of the area of peak d and peak e+e', the functionalization degree of the PEG was determined to be 71%. Further proof came from the FT‐IR analysis, which displayed a peak at 1700 cm^−1^, indicative of the successful introduction of an aldehyde group to PEG (Figure S1, Supporting Information).

### 2.2 Fabrication and Characterization of Gel‐ADH/DF‐PEG Hydrogel

The hydrogel was formed by crosslinking Gel‐ADH with DF‐PEG. While Gel‐ADH provides an abundance of hydrazide groups amenable for bonding, DF‐PEG carries dibenzaldehyde groups, which create aromatic Schiff bases upon bonding. A stable and enduring covalent linkage forms when the hydrazide groups on Gel‐ADH react with aldehydes on DF‐PEG to create hydrazones at neutral pH.

**Figure 3:**
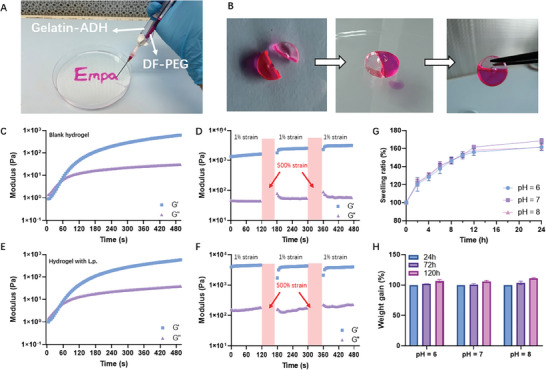
Hydrogel Formation and Characteristics. A) Dual‐syringe system forms a red hydrogel shaped as “Empa”, colored by Rhodamine B. B) Two severed pieces of hydrogel rejoin after being brought into contact and allowed to self‐heal for 2 h. C) Rheology confirms hydrogel formation within 60 s after loading. D) An alternating step strain sweep for the blank hydrogel reveals temporary network collapse at high oscillatory force (*γ* = 500%), swiftly reverting to its initial state (*G*′ ≈ 0.9 kPa). E) ProGel shows similar gelation time and behavior as the blank hydrogel. F) The alternating step strain sweep for ProGel mirrors the temporary collapse and recovery observed in (D), regardless of the addition of probiotics. G) Hydrogels swell to about 160% within 24 h across pH values, 6, 7, and 8. H) After the 24‐h swelling, the hydrogels remain stable in PBS at different pH levels for an additional 4 days. *n* = 3.

Application of this hydrogel can be facilitated through a dual‐syringe system (**Figure** **3**A). The system, with its two distinct cylinders, ensures optimal interaction between the solutions at the outlet, inciting an immediate and controlled crosslinking reaction, allowing the formation of the hydrogel. The process not only supports in situ gelation, obviating the necessity for toxic crosslinkers but also allows to shape the Gel‐ADH/DF‐PEG hydrogel, as evidenced by the facile inscription of “Empa”, attesting to the remarkable injectability of the hydrogel. Compared to ready‐made hydrogels, the injectable form offers a more precise shape control for the administration, which is especially relevant when the hydrogel is applied to treat uneven wounds with irregular and complex shapes.

It is known that the gelation time of injectable hydrogels can be influenced by solid content and component volume ratios.^[^ ^19^ ^]^ Low solid content or slow crosslinking in the hydrogel can extend gelation times, making it unsuitable for wounds due to potential precursor diffusion into the skin. Conversely, high solid content or fast crosslinking can cause too rapid gelation and result in too rigid hydrogels, which is challenging to handle. According to previous research,^[^ ^19^ ^]^ we chose a 4:1 v/v ratio between Gel‐ADH (15% w/v) and DF‐PEG (15% w/v) as the ideal formulation for our hydrogel. The mesh size of the hydrogel was calculated to be ≈ξ = 14 nm using the Flory–Rehner equation^[^ ^22^ ^]^ (Supporting Information). Morphological features of dry samples observed by SEM can be significantly affected by the sample preparation process, such as drying‐induced polymer aggregation and handling‐induced deformation.^[^ ^23^ ^]^ The absence of visible pores in the SEM images (Figure S4, Supporting Information) of the freeze‐dried blank hydrogel and the hydrogel with probiotics (*L. plantarum*) likely indicates shrinkage of the polymer network during the freeze–drying process. This might also be influenced by the high cross‐linking of gelatin. The blank hydrogel shows a smooth and uniform surface at both lower and higher magnifications. In contrast, the hydrogel with probiotics displays a rougher surface with numerous irregular aggregates, particularly visible at higher magnifications. These aggregates are likely debris from the probiotics or probiotic aggregates formed during the freeze–drying process.

We determined the gelation time of the hydrogel using the intersection of the *G*′ and *G*′′ curves, a widely accepted method.^[^ ^19^ ^]^ Rheological testing revealed that gelation occurred ≈60 s after loading the hydrogel onto the rheometer (Figure 3C), indicating an immediate and controlled crosslinking reaction between the two components. The tube inversion method showed a slightly longer gelation time of around 150 s (see Video S1 in the Supporting Information). The rapid gelation of the hydrogel is advantageous in preventing it from spreading to adjacent tissues. The formed hydrogel was washed in water and freeze‐dried for FTIR assay (Supporting Information). In addition to the typical peaks associated with gelatin, peaks characteristic of PEG are also observed. Specifically, the peak at 1142.54 cm^−1^ corresponds to the stretching vibrations of the C–O–C groups, and the peak at 951.77 cm^−1^ is attributed to the CH2 rocking and twisting motions, confirming the presence of PEG in the system. However, the newly formed C═N bonds cannot be distinguished from other existing bonds. Quantification of the cross‐linking degree of the hydrogel was accomplished via the TNBS assay method, revealing a cross‐linking degree of 69 ± 0.37% (Supporting Information), a result comparable to those reported in a previous study.^[^ ^19^ ^]^

Notably, the gel exhibited impressive self‐healing properties arising from the dynamic equilibrium of its Schiff‐base bonds. To visualize this self‐healing property, a hydrogel sample was chopped in half, and the cut pieces were put together. For visualization purposes, one piece of the gel was stained with rhodamine B to differentiate it from another piece (Figure 3B). Upon juxtaposition for ≈1 h, the two pieces seamlessly merged due to the self‐healing characteristics of the hydrogel. Additional analysis, performed through a strain amplitude sweep test, further confirmed this property (Figure 3D). The temporary collapse of the gel network under high oscillatory force (*γ* = 500%) was evidenced by a decrease in the *G*′ value to below the test limit. However, when the amplitude was reduced (*γ* = 1%, frequency = 1.0 Hz), the hydrogel quickly regained its original state (*G*′ ≈ 0.9 kPa), signifying a swift recovery of the internal network structure. The hydrogel's self‐healing properties, derived from dynamic Schiff‐base bonds, allow automatic damage repair via acylhydrazone exchange. Upon mechanical perturbation, these bonds at the impacted site can dissociate and subsequently reform, facilitating spontaneous structural recovery. For wound patches, such self‐healing attributes are paramount. They ensure sustained adaptability to changing wound morphologies and foster continuous interface contact.^[^ ^16^, ^21^ ^]^ This resilience not only mitigates wear but also preserves a conducive moist healing milieu. Furthermore, the self‐regenerative barrier minimizes potential infection pathways and precludes erratic therapeutic agent release, thereby enhancing wound treatment efficiency and reducing the need for frequent patch replacements. Figure 3G shows the normalized swelling ratio (%) of hydrogels over time at pH levels 6, 7, and 8, simulating different physiological conditions: pH 6 for epithelialized wounds, pH 7 as neutral, and pH 8 for alkaline chronic wounds. All conditions rapidly increased to about 160% within 24 h demonstrating hydrogels' robustness across pH levels. Swelling properties are crucial for hydrogels' effectiveness as wound patches because they retain moisture, absorb excess exudate, and promote faster healing.^[^ ^24^ ^]^ After 24 h of swelling, the hydrogels were kept in PBS at different pH levels to test the swelling ratio as a measure of potential degradation. Figure 3H shows that the hydrogels remained stable in the PBS for another 4 d without losing their weight, indicating their stability.

### 2.3 Characterization of *L. Plantarum*‐Entrapping Hydrogels

The hydrogel incorporating live probiotics, namely the ProGel, was prepared by premixing a probiotic suspension with DF‐PEG prior to the crosslinking phase of DF‐PEG. Following this, the mixture was injected using a dual‐syringe system to produce the living hydrogel, a procedure mentioned above. Upon mixing, the two components initiate the gelation process, and within a minute, the gel is formed (*G′ > G″*) (Figure 3F), irrespective of the addition of the probiotic. This suggests that the inclusion of probiotics does not interfere with the gelation process. Similarly, the self‐healing properties of the hydrogels remained even after incorporating the probiotics (Figure 3G). When a high strain (500%) was removed, the hydrogel fully recovered its mechanical strength within seconds. This demonstrates its superior self‐healing property, which could effectively prevent the burst release of entrapped probiotics in the event of an unexpected rupture of the hydrogel.

**Figure 4:**
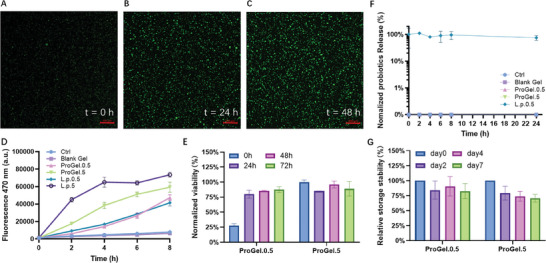
Viability of the Entrapped Probiotics and Their Release from ProGel. A–C) 3D confocal images show probiotic *L. plantarum* embedded in hydrogel at sequential time intervals: immediately postformation (0 h), and at 24 and 48 h [Scale bar: 200 µm]. Increased probiotics density can be observed with time, suggesting compatibility of ProGel with probiotics. D) Viability of entrapped and planktonic *L. plantarum* was assessed with AlamarBlue Assay. Progressive increase in fluorescence intensity confirming the compatibility of ProGel with probiotics. ProGel.0.5 and ProGel.5 denote that the probiotics entrapped in the hydrogel have OD = 0.5 and 5, respectively. E) The viability of the probiotics entrapped in ProGels on ex vivo human skin models (with DMEM (+10% FCS) as nutrition source) at different times. F) Release of probiotics from ProGel. ProGel significantly restricted the release of *L. plantarum*, with no detectable amount being released from the hydrogel within 24 h. G) The temporal viability of the entrapped probiotics in ProGel at lower concentration (OD = 0.5) and higher concentration (OD = 5). *n* = 3 (biological repeats).

The viability of the probiotics entrapped within the hydrogel was further assessed with two methods. Firstly, confocal microscopy analysis of the hydrogels after live/dead staining was performed. As illustrated in **Figure** **4**A, abundant individual probiotic bacteria were visible with green fluorescence, indicating the biocompatibility of the hydrogel and its nontoxicity throughout the preparation process. Comparison of the initial image after gelation (Figure 4A) with subsequent images captured after 24 h (Figure 4B) and 48 h (Figure 4C), respectively, revealed that the individual bacteria form bigger colonies with time. The dramatic increase in cell density further confirms the material's aptness for bacterial growth.

Further quantitative examination of the metabolic activity of the entrapped probiotics was conducted via the AlamarBlue assay. AlamarBlue reagent was added into the Man–Rogosa–Sharpe (MRS) culture medium with the ProGel. As shown in Figure 4D, the hydrogel‐entrapped *L. plantarum* exhibited similar trends to the free ones (i.e., the planktonic *L. plantarum*), showing a typical exponential increase in fluorescence intensity, indicative of bacterial metabolic activity. Notably, the hydrogel‐entrapped probiotics retained over 80% of their viability compared to the same amount of nonentrapped probiotics, confirming not only the compatibility of the hydrogels with the entrapped probiotics but also their permeability for small molecules like resazurin. To further investigate the living hydrogel's viability under more realistic physiological conditions, we conducted viability tests on an ex vivo human skin model with Dulbecco's Modified Eagle Medium (DMEM) (+10% foetal calf serum (FCS) as the nutrient source. As shown in Figure 4E, in a nutrition‐rich wound environment, ProGel.0.5 (entrapped probiotics has OD = 0.5) increased its viability within the first 24 h to the level of ProGel.5 (entrapped probiotics has OD = 5) and remained stable for another 48 h. This increase is likely due to the proliferation of the encapsulated probiotics in ProGel.0.5. In contrast, ProGel.5 maintained high viability without further growth, sustaining its viability for at least 72 h. On the nutrition‐poor ex vivo skin model (Figure S5, Supporting Information), both ProGels maintained their viability for the first 24 h but showed a decrease thereafter. This decline can be attributed to the lack of necessary nutrients for the encapsulated probiotics to remain viable. The storage stability of the ProGels was also tested: 12 identical ProGel samples were prepared and stored at 4 °C. At predetermined time points—Day 0, Day 2, Day 4, and Day 7—triplicate samples (three individual ProGels) were taken from storage and their viability was tested via AlamarBlue assay. Figure 4G shows the temporal viability of ProGels. Initially, on Day 0, the average viability is at its maximum, normalized to 100%. Over time, a gradual decline in viability is observed, with Day 2, Day 4, and Day 7 showing progressively lower mean viabilities. By Day 7, the viability is nearing 70%, indicating that while there is some loss in viability, the majority of the probiotics in ProGel remain viable after a week of storage. Stability upon storage of ProGel ensures its therapeutic effectiveness, safety, and longer shelf life, making it cost‐effective and practical for healthcare use.

Furthermore, the hydrogel effectively limited the uncontrolled release of the entrapped probiotics, as expected based on the calculated mesh size of ≈14 nm. To validate its capacity to prevent bacterial escape, the living hydrogel was immersed in PBS buffer for 24 h, and the bacterial cells released from the hydrogel were quantified by plating the solution on agar every 2 h and consequently counting the colony‐forming units (CFUs). As shown in Figure 4F, the CFU value for nonentrapped probiotics remained steady, indicative of stable probiotic viability in the solution. However, no countable colonies were identified for probiotics entrapped in the hydrogel, suggesting a significant constraint on probiotic release. This restriction of the release of the entrapped probiotics was also confirmed on MRS agar plates and our ex vivo skin model (Supporting Information). The retention efficacy of our hydrogel is largely determined by its mesh size, which tends to reach around 14 nm. Considering that *L. plantarum* dimensions typically range from 0.9–1.2 µm in width and 3–8 µm in length,^[^ ^25^ ^]^ they greatly exceed the mesh size of the hydrogels. This smaller mesh acts as a natural barrier against bacterial movement, limiting the release of entrapped components like probiotics.^[^ ^22^ ^]^ Consequently, these probiotics remain effectively trapped within the hydrogel. Moreover, the intrinsic structure of the hydrogels, including viscosity and tortuosity, may further limit the diffusion of entrapped entities.^[^ ^26^ ^]^

These results affirm the excellent trapping property of the fabricated living hydrogel, thereby mitigating the risk of uncontrolled release of probiotics, which could result in wound contamination. At the same time, the pore size would be large enough to allow free diffusion of nutrients and antimicrobial agents produced by *L. plantarum*.

### 2.4 Antimicrobial Effects of the *L. Plantarum*‐Entrapping Hydrogels

Since ProGel, enriched with entrapped probiotics, was specifically engineered to combat wound infections, it is crucial to assess its antimicrobial activity against prevalent wound pathogens such as *P. aeruginosa*, *S. aureus*, and *C. albicans*. The ubiquity of these pathogens in wound infections, coupled with their notorious resistance to antibiotics or antifungals, and propensity for protective biofilm formation, poses a formidable challenge for innovative therapeutic strategies.

**Figure 5:**
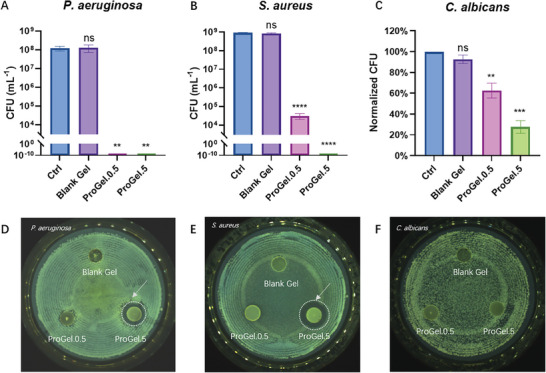
Antimicrobial Efficacy of the ProGels against Common Wound Pathogens. A–C) These panels present the viability of common wound pathogens—*P. aeruginosa*, *S. aureus*, and *C. albicans*—after coincubating for 24 h with hydrogels, either loaded or unloaded with entrapped *L. plantarum*. The ProGel, with a high probiotic concentration (OD = 5), effectively eradicated both bacterial pathogens (*P. aeruginosa*, *S. aureus*, A and B). Even the hydrogel with a lower probiotic load (OD = 0.5) managed to fully eliminate *P. aeruginosa*, while reducing *S. aureus* by roughly 3log CFU. C) The antifungal effect of the ProGels against *C. albicans* was not as strong as its antibacterial power, an elevation in the probiotic load within the hydrogel correlated with augmented antifungal activity, effectuating a reduction of 40% and 70% for a hydrogel with low (OD = 0.5) and high (OD = 5) probiotic loads, respectively. D–F) The agar diffusion assay demonstrates the hydrogel's antimicrobial properties against the mentioned pathogens. Notably, clear inhibition zones (indicated with the arrows and dotted circle) are visible against bacterial pathogens with the ProGels (D and E), especially those with higher probiotic loads. However, no significant inhibition zones were observed for *C. albicans* (F). *** denotes statistical difference (*P* < 0.001) and ** (*P* < 0.01) using the ANOVA test. *n* = 3 (biological repeats), mean ± SD shown.

In vitro antimicrobial tests with ProGel hydrogels, both with and without embedded probiotics, were performed against these pathogens. While the ProGels with a high probiotic load (OD = 5, ≈10^8^ CFU mL^−1^) successfully killed the two bacterial pathogens completely (i.e., *P. aeruginosa*, *S. aureus*, **Figure** **5**A,B), the hydrogel with a lower probiotic load (OD = 0.5) could still fully eliminate *P. aeruginosa* but only reduced *S. aureus* by ≈3log CFU. This difference in efficiency may be attributed to the higher susceptibility of *P. aeruginosa* to antimicrobial substances produced by *L. plantarum*, particularly lactic acid, which lowers the local pH, creating an unfavorable environment for bacterial growth. In contrast, *S. aureus* is known to exhibit pH resilience, tolerating a pH range from 4.2 to 9.3.^[^ ^27^ ^]^ Other factors such as plantaricin, a bacteriocin produced by *L. plantarum*, might play a more important role in the antibacterial activity against *S. aureus*,^[^ ^28^ ^]^ contributing to the observed reduction of viable *S. aureus*. Interestingly, although the living hydrogel's antifungal effect against *C. albicans* was not as strong as its antibacterial effects, still, enhanced antifungal activity was found for hydrogels with higher amount of probiotic (Figure 5C), achieving a ≈40% and ≈70% viability reduction for hydrogel with a low (OD = 0.5) and high (OD = 5) probiotic load, respectively. The pronounced effect observed contrasts with a prior study suggesting that *L. plantarum* did not directly inhibit the proliferation of *C. albicans*.^[^ ^29^ ^]^ This divergence may be attributed to the augmented presence of viable probiotics within our living hydrogel. Given that the prior release test indicated significant restriction of probiotic release from the hydrogel, it is plausible that the observed antimicrobial effects largely rely on compounds secreted by the probiotics rather than direct cell‐to‐cell contact. This hypothesis aligns with prior studies utilizing probiotics to treat pathogen biofilms.^[^ ^30^ ^]^

To further validate our findings, we conducted an agar diffusion assay, which mimics more the wound environment than the co‐incubation in solution. Clear inhibition zones were observed for bacterial pathogens with the ProGel (Figure 5D,E), particularly those with higher probiotic loads. However, no inhibition zones were observed for *C. albicans* (Figure 5F), affirming the less pronounced results from the co‐incubation tests.

### 2.5 The Cyto‐ and Hemo‐Compatibility of the Hydrogels

**Figure 6:**
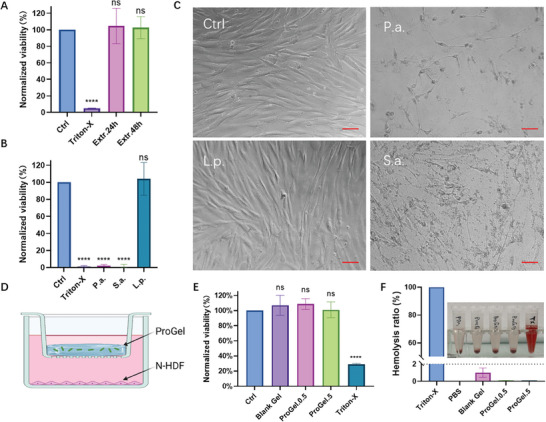
Cytotoxicity and hemocompatibility of ProGel. A) Hydrogel extracts (24 and 48 h) maintained high cell viability, indicating excellent cytocompatibility. B) nHDFs cocultured with *L. plantarum* showed no cytotoxicity, unlike those with *P. aeruginosa* and *S. aureus*, confirmed by the CCK‐8 assay. C) Microscopy shows detached, round dead cells in cultures with *P. aeruginosa* and *S. aureus*, while cells with *L. plantarum* remained intact. Scale bar = 100 µm. D) A transwell (0.4 µm pore size) allowed the separation of components, ensuring signals from nHDFs only. E) CCK‐8 assay results show no cytotoxicity from the living hydrogel after one day of coculture. F) Hemolysis assay results indicate all tested materials, except the Triton‐X control, had hemolysis rates below the 2% safety threshold, confirming hemocompatibility. *** denotes statistical significance (*P* < 0.0001) using ANOVA test. *n* = 3 (biological repeats), mean ± SD shown.

Cytocompatibility is essential for wound dressings. To evaluate the cytotoxicity of the hydrogel and its potential degradation products, normal human dermal fibroblasts (nHDFs) were cocultured with hydrogel extracts. **Figure** **6**A shows that both the 24 and 48 h hydrogel extracts maintained high cell viability compared to the negative and positive controls, indicating excellent cytocompatibility. For biocompatibility assessment of probiotics compared to pathogens, *L. plantarum, P. aeruginosa, and S. aureus* were separately cocultured with nHDFs. *P. aeruginosa* and *S. aureus* inoculated with nHDFs exhibited significant cytotoxicity (Figure 6B), whereas nHDFs cocultured with *L. plantarum* remained unaffected for up to 24 h, as confirmed by the Cell Counting Kit‐8 (CCK‐8) quantitative assay. Morphological examination under microscopy showed detached, round dead cells in cultures with *P. aeruginosa* and *S. aureus* (Figure 6C), while cells cocultured with *L. plantarum* remained intact, similar to the control. This observation aligns with the results from our recent study where nHDFs remained unaffected after 24 coculture with a lactobacilli cocktail.^[^ ^30^ ^]^ The overall cytocompatibility of the living hydrogel system, ProGel, was evaluated by coculturing nHDFs with the hydrogel (Figure 6D). To ensure the CCK‐8 viability signal was solely from nHDFs, a transwell system (0.4 µm pore size) was used to separate the hydrogels from the cells, preventing any physical interference from the hydrogel or noise signal from the entrapped probiotics.^[^ ^31^ ^]^ Compared to the negative control (DMEM medium), both blank hydrogels and ProGel with low and high probiotic loads showed no cytotoxicity towards nHDFs (Figure 6E), confirming the biological safety of ProGel and underscoring its potential for safe topical use without adverse cytotoxic reactions.

Given the hydrogel's intended application on wounds where it may directly interact with blood, its hemocompatibility is crucial. Hemolysis assays were conducted to assess this property. The figure illustrates that the supernatant in the TritonX group was red, indicating complete hemolysis occurred. In contrast, the supernatants from the other experimental groups remained clear and transparent, showing no signs of hemolysis visible to the naked eye. The hemolysis ratio data presented in Figure 6F demonstrates that all tested materials, i.e., Blank Gel, Pro‐Gel.0.5, and ProGel.5 maintain hemolysis rates significantly below the 2% threshold deemed safe for blood‐contacting biomaterials.^[^ ^15c^ ^]^ This result underscores the hemocompatibility of our probiotic‐laden hydrogels.

### 2.6 Ex Vivo Evaluation of Antibiofilm Activity of the Hydrogels

**Figure 7:**
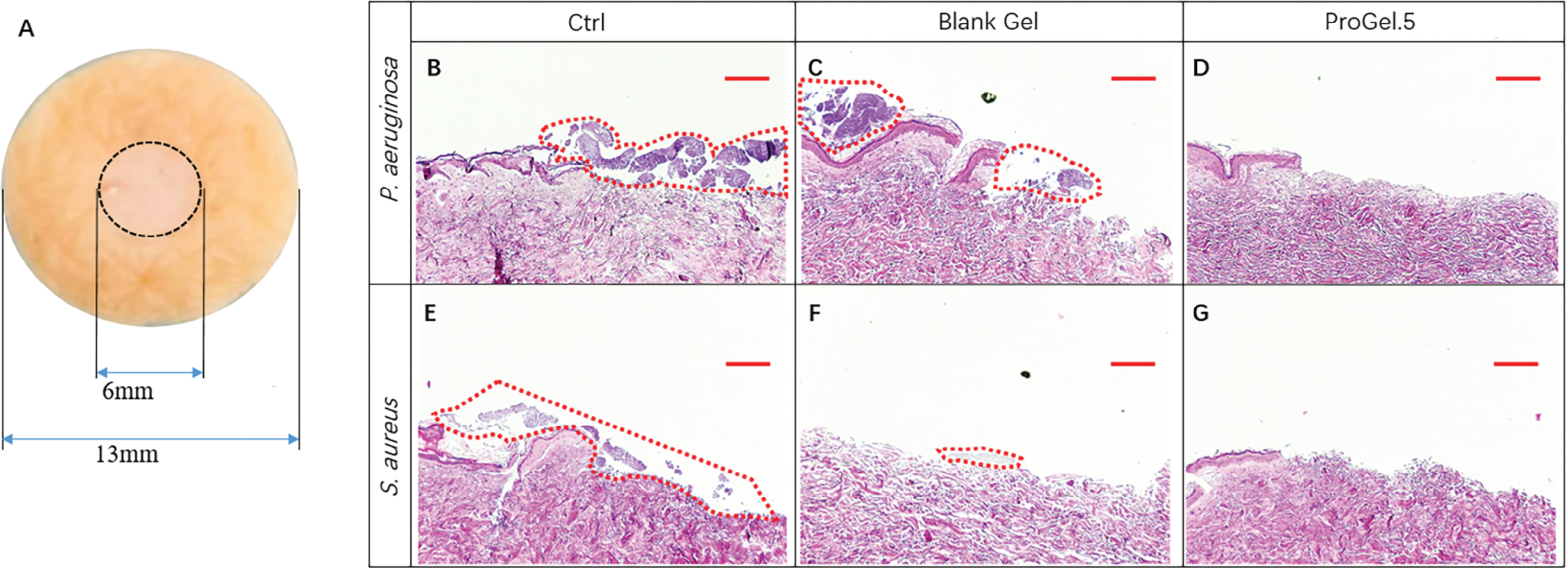
A) Histological Analysis of the Antibiofilm Efficacy of Hydrogels Tested with an Ex vivo Skin Model. Artificial wounds of 6 mm diameter were created on human ex vivo skin samples of 13 mm diameter (A), followed by deliberate infection with *P. aeruginosa* (OD = 0.1) or *S. aureus* (OD = 0.5). Both unloaded and *L. plantarum* loaded hydrogel samples were applied on these infected wounds and subjected to treatment for 24 h (*P. aeruginosa*) and 48 h (*S. aureus*). Panels (B‐D) feature skin samples post‐*P. aeruginosa* infection, B) untreated, C) treated with blank hydrogel, and D) *L. plantarum* loaded hydrogel. Panels (E–G) correspondingly display skin samples post‐*S. aureus* infection, E) untreated, F) blank hydrogel treated, and G) *L. plantarum* loaded hydrogel treated. In the untreated groups, prominent clusters of B) *P. aeruginosa* and E) *S. aureus* biofilms were discernible on the dermis. Notably, while the blank hydrogel significantly F) curbed *S. aureus* biofilm formation, C) its effects against *P. aeruginosa* biofilms were inconsequential. With the ProGel treatment, the presence of biofilms was virtually absent (D, G), thereby reinforcing the potency of the living hydrogel against both pathogens. [Scale bar = 200 µm].

To investigate the antibacterial attributes of the ProGel hydrogel in a context more resembling wound infection, we employed ex vivo human skin models. Compared to common tests on animal models like mice or rabbits, the inherent structural, biochemical, and physiological characteristics of human skin render it particularly suited for such evaluations.^[^ ^32^ ^]^ Artificial wounds (*d* = 6 mm) were introduced on ex vivo human skin samples, which were then intentionally infected with *P. aeruginosa* or *S. aureus* (**Figure** **7**A). Subsequently, these infected wounds were subjected to treatment with hydrogel preparations loaded with varying amounts of probiotics. Infected wounds devoid of any hydrogel treatment served as the negative control. Hematoxylin and eosin (H&E) assay illustrated epidermal disruption due to mechanical wounding (Figure 7B–G). In the untreated groups, clumps of *P. aeruginosa* (Figure 7B) and *S. aureus* biofilms (Figure 7E) were clearly visible on the dermis. Interestingly, while the hydrogel without probiotics showed no substantial effects against *P. aeruginosa* biofilms (Figure 7C), it seemed to inhibit the formation of *S. aureus* biofilms, albeit with a small amount of residual bacterial clumps (Figure 7F). Such outcomes might be attributed to the direct interaction of the hydrogel with the wound, as hydrogels are known to naturally absorb wound exudate and its physical presence could also limit the formation of bacterial biofilms.^[^ ^33^ ^]^ Remarkably, the application of the ProGel led to clear prevention of biofilm formation of both *P. aeruginosa* and *S. aureus* (Figure 7D,G), thereby reaffirming the effectiveness of the ProGel against both pathogens. This result highlights the living hydrogel's substantial potential as an excellent antibacterial material in wound care.

## 3 Conclusion

This research presents ProGel, an injectable gelatin‐based living hydrogel loaded with probiotic *L. plantarum*, as a therapeutic agent for the treatment of skin wound infections. Notably, it exhibits strong mechanical properties and self‐healing capabilities due to its Schiff‐base bonds, countering potential hydrogel rupture. Furthermore, ProGel effectively preserves the viability of the entrapped *L. plantarum* after the gelation process, maintaining over 80% survivability while preventing probiotic escape. In addition to its robust design and probiotic retention, ProGel demonstrates broad‐spectrum antimicrobial efficacy against primary wound‐associated pathogens—*P. aeruginosa*, *S. aureus*, and *C. albicans*. Its antimicrobial effectiveness has been corroborated through tests on a human ex vivo skin wound model infected by *P. aeruginosa* and *S. aureus*. It showed high cytocompatibility with human dermal fibroblasts, marking it as a safe and effective option for wound care. The ProGel serves potentially as an innovative platform for integrating other living organisms to expand the scope of bioactive effects, including but not limited to modulation of the wound microenvironment and enhanced wound healing.

## 4 Experimental Section

The materials and reagents, including PEG2000, 4‐formylbenzoic acid (97%), 4‐dimethylaminopyridine (DMAP, ≥99.0%), tetrahydrofuran (THF, anhydrous, ≥99.9%), diethyl ether (≥97.5%), gelatin (from porcine skin, Type A), adipic acid dihydrazide (ADH, ≥98%), 1‐hydroxybenzotriazole (HOBt, ≥97.0%), dimethyl sulfoxide (DMSO, ≥97%), *N,N'*‐dicyclohexylcarbodiimide (DCC), *N*‐ethyl‐*N*′‐(3‐dimethylaminopropyl)carbodiimide hydrochloride (EDC·HCl), 2,4,6‐trinitrobenzenesulfonic acid (TNBS), and sodium bicarbonate, were procured from Sigma‐Aldrich (Buchs, Switzerland) and used as received. *L. plantarum* (ATCC 10 241), *P. aeruginosa* (ATCC 43 390), *S. aureus* (ATCC 6538), and *C. albicans* (ATCC 90 028) were purchased from the Leibniz Institute DSMZ. The human skin samples for ex vivo study were kindly provided by the Cantonal Hospital St. Gallen. The skin samples were surplus materials from routine surgeries with informed consent from all patients involved. This study was exempted from ethical approval.

### Aldehyde Modification of PEG

The aldehyde modification of PEG was conducted based on established protocols.^[^ ^21b^ ^]^ Initially, PEG2000 (3.26 g, 1.63 mmol), 4‐formylbenzoic acid (0.98 g, 6.52 mmol), and DMAP (0.050 g) were dissolved in 100 mL of anhydrous THF. Subsequently, DCC (1.68 g, 8.15 mmol) was introduced under a nitrogen atmosphere. The mixture was stirred continuously at 20 °C for 18 h. The formed white precipitate was isolated and discarded after filtration. Cold diethyl ether was used to precipitate the resultant filtrate, which was then subjected to three consecutive cycles of dissolution in THF and precipitation in diethyl ether to ensure purity. After the purification process, the white solid was desiccated under vacuum. The characterization of the final product, i.e., the DF‐PEG was accomplished using ^1^H NMR (Bruker 500 MHz) and ATR–FTIR (Bruker Alpha) spectrometry.

### Gel‐ADH Synthesis and Characterization

Gel‐ADH was synthesized following an established protocol.^[^ ^34^ ^]^ Gelatin (3 g) was dissolved in MilliQ water (300 mL) at 40 °C, to which ADH (2.2 g, 12.6 mmol) was subsequently added at ambient conditions. Concurrently, solutions of HOBt (0.45 g, 3 mmol) in DMSO (10 mL) and EDC (0.46 g, 2.4 mmol) in H_2_O (10 mL) were prepared and combined. This activator mixture was then added dropwise to the gelatin solution, with the pH subsequently adjusted to 5.0 using dilute HCl. After 24 h of stirring at room temperature, the product underwent a sequential dialysis at 40 °C: initially against a 0.3 m NaCl solution for two days, followed by a 25% v/v ethanol solution for two days, and culminating with MilliQ water for final two days, employing a dialysis membrane with MWCO of 6–8 kDa. The modified gelatin, designated as Gel‐ADH, was retrieved postlyophilization and characterized using ^1^H NMR. The grafting ratio, indicating the substitution rate of carboxy groups with hydrazide groups, was determined via the TNBS assay (supportive information).^[^ ^34^ ^]^

### Preparation of Hydrogels with/without Entrapped Probiotics

The probiotic strain *L. plantarum* was first cultured in MRS broth and incubated under agitation (160 rpm) at 37 °C overnight. Subsequently, it was washed twice with and resuspended in phosphate‐buffered saline (PBS, pH = 7.4) to yield final optical densities (OD) of ≈50 or 5, corresponding to diluted OD values of 0.5 at 100‐fold or tenfold dilutions, respectively. The *L. plantarum* stock was then mixed with a 30% w/v DF‐PEG solution (prepared in PBS) to form precursor A, which was then loaded into barrel A of the dual‐syringe system. For hydrogels void of *L. plantarum*, an equivalent volume of PBS replaced the probiotic stock in precursor A. Concurrently, a 15% w/v Gel‐ADH solution in PBS was prepared as precursor B and filled into barrel B. The cross‐sectional area ratio of barrels A to B was set at 1:4. The designated ProGel (with probiotics) or blank Gel (without) were fabricated by coextruding precursor A and B at a volume ratio of 1:4 through a static mixer connected to the outlets of the two barrels, followed by immediate deposition into a silicone mold (*d* = 6 mm). This process induced swift in situ gelation to form a hydrazone crosslinked hydrogel. The mixture was retained in the mold for 1 h to ensure complete gelation. The gelation was confirmed using the tube inversion method, where Gel‐ADH solution and DF‐PEG were mixed in a glass vial and inverted. The formed hydrogel is washed in water and freeze‐dried for FTIR assay. Given that the probiotic stock comprises 10% of the final mixture's volume, the resultant ProGel features a tenfold diluted probiotic concentration. Thus, a ProGel formulated with an *L. plantarum* stock of OD ≈ 50 yields a final OD ≈ 5, denoted as ProGel.5. Conversely, a ProGel derived from an *L. plantarum* stock of OD ≈ 5 results in an OD ≈ 0.5, labeled as ProGel.0.5. The degree of cross‐linking in the hydrogels was assessed by calculating the relative reduction of amine groups in the crosslinked hydrogel via an adapted TNBS assay method (Supporting Information).

The swelling behaviors and weight loss of the hydrogels were evaluated using a gravimetric method. Hydrogel samples were immersed in 3 mL of PBS at three different pH levels (pH 6, 7, and 8) in a 12‐well plate. These pH levels were chosen to simulate various physiological conditions: pH 6 to represent epithelialized wounds, pH 7 as a neutral pH at acute wounds, and pH 8 to simulate the alkaline pH of some chronic wounds.^[^ ^35^ ^]^ Excess surface water was removed before weighing the samples. At specified time intervals, the hydrogels were removed, wiped, and weighed. The swelling ratio was calculated as *(W*_t_ */W*_0_) × 100%, where *W*_t_ is the weight of the hydrogel at a specific time point t, and *W*_0_ is the initial weight of the hydrogel at 0 h.

### Rheological Analysis of Hydrogel Properties

Rheological evaluations were executed using a rheometer (Anton‐Paar MCR 301). Precursors A and B, combined in a 1:4 volume ratio, were introduced onto the rheometer plate to a total volume of 800 µL. The upper plate (plain plate, diameter 25 mm) was subsequently adjusted to establish a 1 mm gap for assessments. The recording of gelation kinetics started ≈60 s after mixing the precursors and the loading step. The gelation kinetics of the hydrogels were probed using a time sweep test, wherein the storage modulus (*G′*) and loss modulus (*G″*) were recorded as functions of time at a fixed frequency of 1.0 Hz at strain 1%. Subsequently, the hydrogel's self‐healing properties were characterized via a step strain sweep approach, alternating between strains of 1% for 120 s and 500% for 60 s, at a consistent frequency of 1.0 Hz.

### SEM Evaluation of the Hydrogel

Scanning electron microscopy (SEM) images were obtained using a JEOL Hitachi S‐4800 at an accelerating voltage of 2 keV. The hydrogels were first lyophilized and then sliced into thin sections. These sections were coated with a 7 nm layer of Au–Pd. The SEM was then used to observe the morphological structure of the hydrogels and the loaded probiotics.

### Microscopy Evaluation of the Viability of Probiotics Entrapped in Hydrogel

3D confocal images were acquired to visualize *L. plantarum* within the hydrogel matrix at specific intervals: immediately after formation (0 h), 24 h, and 48 h. The ProGel samples were formulated by dispensing 200 µL of the precursor solution into individual wells of a 24‐well plate. Subsequent incubation was performed in MRS medium at 37 °C. To assess cell viability and proliferation within the gels, they were stained using SYTO9 (Thermo Fisher Scientific). Confocal microscopy was utilized to obtain z‐stacks spanning the hydrogel's depth, confirming a consistent cellular distribution.

### Metabolic Activity of Entrapped Probiotics Using AlamarBlue Assay

The metabolic activity of the entrapped probiotics was assessed quantitatively using the AlamarBlue HS assay (Thermo Fisher Scientific).^[^ ^15d^ ^]^ For this study, 100 µL of AlamarBlue HS reagent and 100 µL of probiotics‐laden hydrogels were introduced into 800 µL of MRS medium, followed by incubation at 37 °C under agitation. Concurrently, 100 µL of blank hydrogels served as the negative control, while 100 µL of probiotic suspension (OD ≈ 0.5 and OD ≈ 5) acted as the positive control. The assay measures the conversion of nonfluorescent resazurin to fluorescent resorufin, facilitated by the inherent reducing capacity of viable *L. plantarum*. At 2 h intervals, the emitted fluorescence intensity at 585 nm was recorded, offering a direct measure of the metabolic activity of the entrapped probiotics. In the investigation of storage stability, 12 identical ProGel specimens were prepared and subsequently preserved at 4 °C. Viability assessments were conducted at pre‐determined temporal intervals: Day 0, Day 2, Day 4, and Day 7. During these intervals, sets of triplicate samples were taken from the storage environment for analysis. The viability of these samples was quantitatively evaluated using the AlamarBlue assay.

### Release of Probiotics from the Hydrogel

For the release assay, 100 µL of ProGel was washed and then submerged into 3 mL PBS. In this evaluation, two control sets were included: 100 µL of blank hydrogels were employed as the negative control, highlighting the absence of probiotics, while two distinct concentrations of probiotic suspensions, OD ≈ 0.5, each of 100 µL volume, served as positive controls, representing the maximum release potential. At two‐hour intervals, an aliquot of suspension of 20 µL is taken out and serially diluted on PC agar. The CFU is measured after overnight cultivation.

### In Vitro Antimicrobial Assay of the Probiotics‐Loaded Hydrogels

The antimicrobial potency of ProGel was examined against prevalent wound‐associated pathogens: Gram‐negative *P. aeruginosa*, Gram‐positive *S. aureus*, and the fungal strain *C. albicans*.

A single bacterial or fungal colony from the respective agar plate stock was cultured overnight in 10 mL Tryptic Soy Broth (TSB) media in a 15 mL Falcon tube at 37 °C with agitation (160 rpm). The resulting suspension was centrifuged, the pellet washed and then resuspended in PBS to achieve the target concentration. For the qualitative agar diffusion assay,^[^ ^36^ ^]^ 100 µL of each pathogen suspension of 10^6^ CFU mL^−1^ (OD600 ≈ 0.01 for *S. aureus* and *P. aeruginosa*; OD600 ≈ 0.1 for *C. albicans*) was evenly spread across PC agar plates. 100 µL of either the ProGel or the probiotic‐absent controls were introduced onto these plates. After incubating at 37 °C overnight, the resultant inhibition zones were visualized and documented photographically. Quantitative in vitro antimicrobial assessment involved coculturing pathogens with hydrogels. ProGel or the probiotic‐absent controls were submerged in 800 µL of a TSB‐MRS media combination (1:1 TSB to MRS ratio, to provide the nutritional requirements of both the probiotics and pathogens). A 10 µL portion of the pathogen stock, ≈10^7^ CFU mL^−1^ (OD600 of 0.1 for both *P. aeruginosa* and *S. aureus*, and 1 for *C. albicans*) was added. This mixture was incubated at 37 °C for 24 h under agitation. Postincubation, 20 µL samples were serially diluted in PBS and plated on PC agar. CFU was quantified by a colony counter (Scan 300, Interscience, France).

### Cytotoxicity of the Hydrogels

The cytocompatibility of the living hydrogel system was evaluated using normal human dermal fibroblasts (nHDFs, PromoCell) through three methods: culturing nHDFs with hydrogel extracts, direct co‐culture with planktonic bacteria, and direct co‐culture with the living hydrogel system. Postincubation, cell viability was assessed using CCK‐8 with untreated cells as 100% viability and cells treated with 1% Triton X‐100 as the positive control. nHDFs were maintained in T‐75 flasks with DMEM supplemented with 10% FCS and incubated at 37 °C with 5% CO_2_.

For cytotoxicity Hydrogel and its potential degradation products, hydrogel extracts were prepared in DMEM with 10% FCS at 1 mg mL^−1^ and incubated at 37 °C with 100% humidity and 5% CO_2_ for 24 and 48 h. nHDFs were seeded at 10 000 cells per well in 100 µL DMEM with 10% FCS one day prior to incubation with extracts. To compare the cytocompatibility of relevant bacteria, *L. plantarum, P. aeruginosa*, and *S. aureus* were cocultured with nHDFs at an equal amount (i.e., at a multiplicity of infection of 1).^[^ ^30^ ^]^ nHDFs were seeded at 10 000 cells per well in 96‐well plates, inoculated with bacteria, and incubated for 24 h. Microscopy images were taken to document cell morphology (Zeiss Primovert, 10× magnification). Then the cells were washed with PBS to remove the coculturing bacteria before assessing viability with CCK‐8. Groups with only bacteria without cells are used as the background for the corresponding bacteria‐cell coculture group, to remove the viability noise generated by the potentially remaining bacteria after wash. For the living hydrogel system, nHDFs were seeded at 20 000 cells per well in 12‐well plates and cocultured with hydrogels using a transwell system to prevent physical contact.^[^ ^31^ ^]^ After 24 h, cell viability was measured using CCK‐8, with untreated cells as the negative control and 1% Triton X‐100 treated cells as the positive control.

### Hemolysis Assay

Human whole blood was obtained from healthy donors (males and females, healthy, ≥18 years, ethical approval BASEC Nr. PB_2016‐00816 from the local ethics committee) by venipuncture technique using 9 mL S‐Monovette tubes (Sarstedt, Switzerland). To prepare the blood samples, 1 mL of whole human blood was anticoagulated with one drop of saturated disodium EDTA solution, followed by centrifugation at 3500 rpm for 15 min to isolate the erythrocytes. These erythrocytes were washed three times with PBS and resuspended in 9 mL of PBS. For the experiment, 200 µL of hydrogel sample was added to 1 mL of the erythrocyte suspension and incubated at 37 °C for 2 h. Positive controls used Triton‐X100 to induce full lysis, while negative controls used PBS to ensure no lysis. Postincubation, the samples were centrifuged, photographed, and the erythrocytes were examined under a microscope. Optical density (OD) of 100 µL supernatant was measured at 545 nm to calculate the hemolysis rate (HR) using the formula HR(%) *= (D*_h_ *−D*_p_ *)/(D*_t_ *−D*_p_ *)×*100%, where *D*_h_ ​, *D*_p_, and *D*_t_ are the absorbance of the supernatant treated by the hydrogels, PBS, and Triton‐X100 (1%), respectively.

### Ex Vivo Evaluation of Antibiofilm Activity of the Hydrogels

Ex vivo human skin models, procured from Cantonal Hospital St. Gallen with informed and anonymous donor consent (exempted from ethical approval), were utilized to evaluate the antimicrobial performance of ProGel.^[^ ^36^ ^]^ Skin sections, ≈13 mm in diameter, were prepared, and artificial wounds of about 6 mm in diameter were generated using a mechanical punch.^[^ ^30^ ^]^ For disinfection, the samples were treated with Octenisept (Schülke & Mayr GmbH, Norderstedt, Germany) for 1 min, followed by three successive washes in sterile PBS, each lasting 10 min. These prepared samples were then placed in 12‐well plates containing 200 µL DMEM. To induce infections, 10 µL of either *P. aeruginosa* (OD600 = 0.1, in TSB medium) or *S. aureus* (OD600 = 0.5, in TSB medium) were inoculated onto the artificial wounds. After an hour, 100 µL of either ProGel.5 or blank gel was applied to the wound surfaces as treatment. The samples were then incubated at 37 °C for 24 h for *P. aeruginosa* and 48 h for *S. aureus*. Wounds that remained untreated served as negative controls.

Histological evaluations were carried out to analyze pathogen infection and biofilm formation on the wounds, with and without hydrogel intervention.^[^ ^36^ ^]^ Post incubation, the skin samples were immersed in 4% formalin and stored at ambient temperature for fixation. These skin specimens were dehydrated through an ethanol gradient to xylene, embedded within paraffin blocks, and sectioned to 5 µm thickness, followed by mounting on glass slides. Hematoxylin and eosin (H&E) staining was conducted in accordance with the manufacturer's instructions. Imaging was performed using a Leica DM4000 B LED microscope.

### Statistic

Experiments were performed in biological triplicates, and data are presented as mean ± standard deviation (SD). Bacterial CFU counts were logarithmically transformed prior to statistical analysis. The viability of entrapped probiotics at *t* = 0 was normalized to 100%. In the cytotoxicity test, the cell viability of the control group was normalized to 100%, and the hemolysis ratio of Triton‐X was also normalized to 100%. Differences between groups were determined using one‐way ANOVA followed by Dunnett's test for multiple comparisons. Statistical analysis was conducted using GraphPad Prism software. Statistical significance was denoted as ***P < 0.001 and **P < 0.01. Prism Graphpad was used for data analysis.

### Ethics Approval

Human whole blood was obtained from healthy donors (males and females, healthy, ≥ 18 years, ethical approval BASEC Nr. PB_2016‐00816 from the local ethics committee). The human skin samples for ex vivo study were kindly provided by the Cantonal Hospital St. Gallen. The skin samples were surplus materials from routine surgeries with informed consent from all patients involved. This study was exempted from ethical approval.

## Conflict of Interest

The authors declare no conflict of interest.

## Author Contributions

S.T.: Conceptualization, Methodology, Validation, Formal analysis, Investigation, Visualization, Writing – Original Draft, Writing – Review and Editing. S.Z.: Methodology, Investigation, Validation. K.W.: Methodology, Investigation, Validation, Writing – Review and Editing. K.M.‐W.: Supervision, Resources, Writing – review and editing. Z.L.: Resources, Writing – review and editing. Q.R.: Conceptualization, Funding acquisition, Methodology, Validation, Supervision, Project administration, Writing – Review and Editing.

## Supporting information

Supporting Information

Supplemental Video 1

## Data Availability

The data that support the findings of this study are available from the corresponding author upon reasonable request.
