# Supplementary material for: An Injectable Living Hydrogel with Embedded Probiotics as a Novel Strategy for Combating Multifaceted Pathogen Wound Infections
Source: Adv Healthc Mater. 2024 Jul 14;13(27):2400921. doi: 10.1002/adhm.202400921 (PMC12344615; doi:10.1002/adhm.202400921)
Supplement: Supplementary file 1 — Supporting Information [file ADHM-13-0-s001.docx]

Supporting information


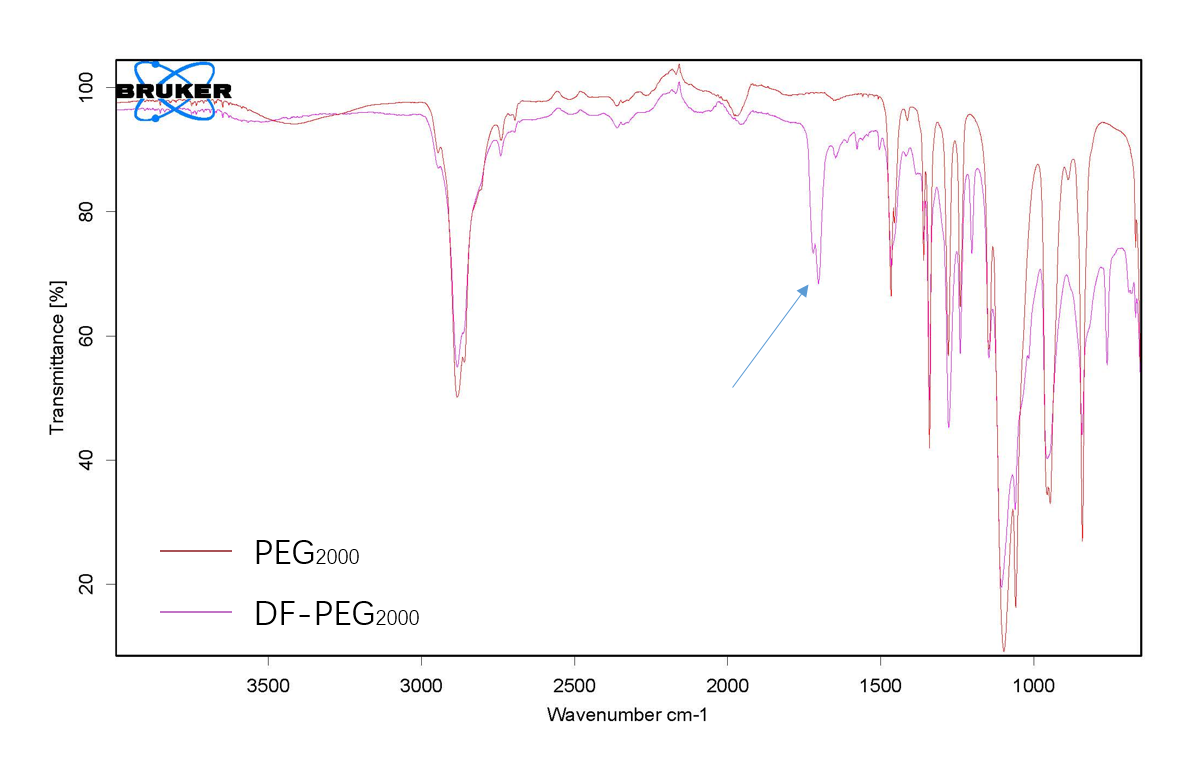


**Figure S1**: FT-IR spectra of DF-PEG and PEG_2000_. The peak at 1700 cm^−1^ further indicate the successful introduction of an aldehyde group to PEG.


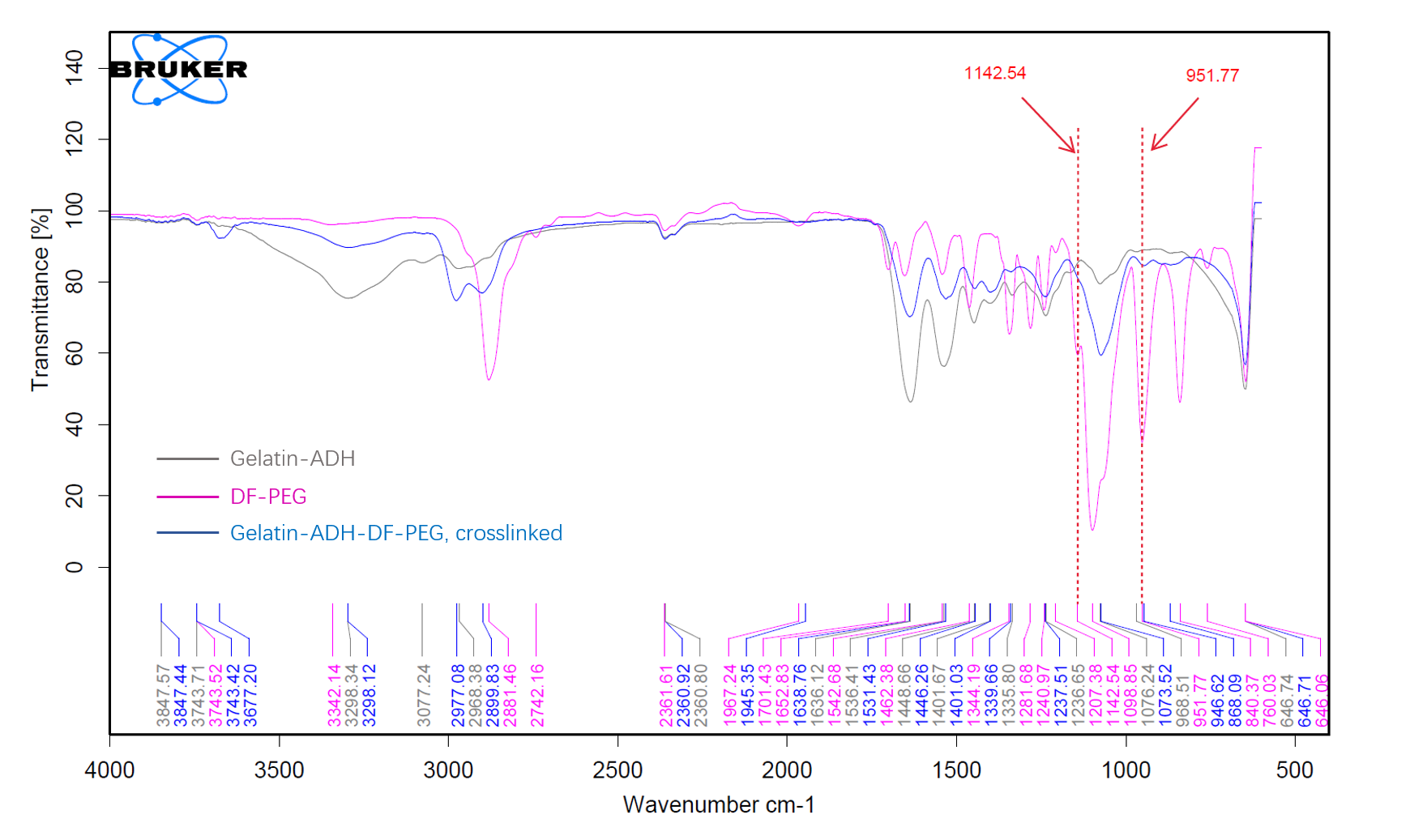


Figure S2. FT-IR spectra of DF-PEG, Gelatin-AHD and crosslinked hydrogel (Gelatin-ADH + DF-PEG). In addition to the typical peaks associated with gelatin (e.g. 1636 cm^–1^, 1536 cm^–1^ and 1236 cm^–1^ for amides), peaks characteristic of PEG are also observed. Specifi-cally, the peak at 1142.54 cm^-1^ corresponds to the stretching vibrations of the C-O-C groups, and the peak at 951.77 cm^-1^ is attributed to the CH2 rocking and twisting motions, confirming the presence of PEG in the system. However, the newly formed C=N bonds cannot be distinguished from other existing bonds.

# TNBS assay

The degree of ADH-substitution, also known as the Grafting Ratio (GR) of the gelatin, i.e. the substitution rate of carboxy groups with hydrazide groups on gelatin, was quantitatively evaluated via the well-established TNBS assay [1], as detailed in Equation 1. A calibration curve (Figure S2) was generated using a series of β-alanine concentrations (0, 0.01, 0.02, 0.04, 0.08, and 0.1 mmol) each dissolved in 0.1 M NaHCO_3_. Each 0.5 ml aliquot of β-alanine solution was reacted with 1 ml of 0.1% w/v 2,4,6-Trinitrobenzenesulfonic acid in 0.1 M NaHCO_3_, followed by a two-hour incubation at 37°C. Post-incubation, the reaction mixtures were treated with 0.25 ml of 10% w/v sodium dodecyl sulfate and 0.125 ml of 1 M hydrochloric acid. Absorbance values at 349 nm were subsequently recorded. To quantify the amine groups in gelatin and gelatin-ADH, 0.1 mg/ml solutions of each were prepared in distilled water and subjected to the TNBS assay. The amine group concentration was ascertained by referencing the absorbance readings to the established β-alanine calibration curve. The amount of carboxylic acid in gelatin is considered 0.8 mmol/g [2].

$$\begin{aligned} GR = \frac{mol conc. of amine groups in gel_{ADH}- mol conc. of amine groups in gel}{mol conc. of carboxy groups in gel}*100 \% \#\left( 1 \right) \end{aligned}$$


**Figure S3**: Calibration curve generated using various known concentrations of β-Alanine.

The degree of cross-linking in the hydrogels was assessed by calculating the relative reduction of amine groups in the crosslinked hydrogel via an adapted TNBS assay method [1]. 2 mg of lyophilized crosslinked-hydrogel (gelatin-ADH + DF-PEG) and its non-crosslinked counterpart (gelatin-ADH + PEG, serving as the control group) were separately combined with 1 ml of 4% w/v NaHCO₃ and 1 ml of 0.05% w/v 2,4,6-Trinitrobenzenesulfonic acid in 0.1 M NaHCO₃. These solutions were incubated at 40°C for 4 hours. Subsequently, 3 ml of 6 M hydrochloric acid was introduced, and the mixtures were further incubated at 40°C for another 4 hours. Absorbance values at 349 nm were obtained. The degree of cross-linking was then derived from the provided equation.

$$\begin{aligned} Crosslinking degree = 1-\frac{{Abs}_{crosslinked-hydrogel}}{{Abs}_{noncrosslinked-hydrogel}}*100 \% \#\left( 2 \right) \end{aligned}$$

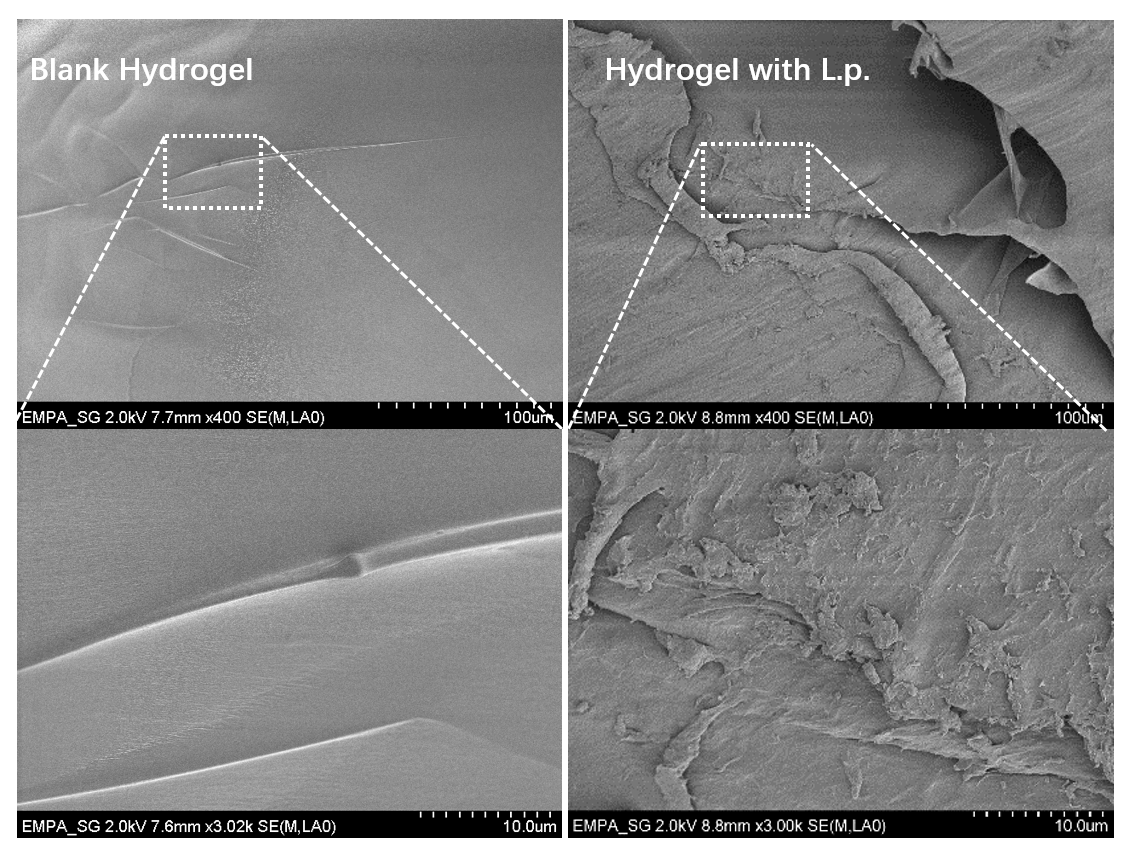


**Figure S4.** SEM images show non-porous structures in both blank and probiotic hydrogels due to high cross-linking of gelatin and polymer shrinkage during freeze-drying

**Figure S5.** Viability of Probiotics Entrapped in ProGel on Ex Vivo Skin Samples Moistened with PBS. In the nutrition-poor ex vivo skin model, both ProGels maintained their viability for the first 24 hours but showed a decrease thereafter. This decline can be attributed to the lack of necessary nutrients for the encapsulated probiotics to remain viable.


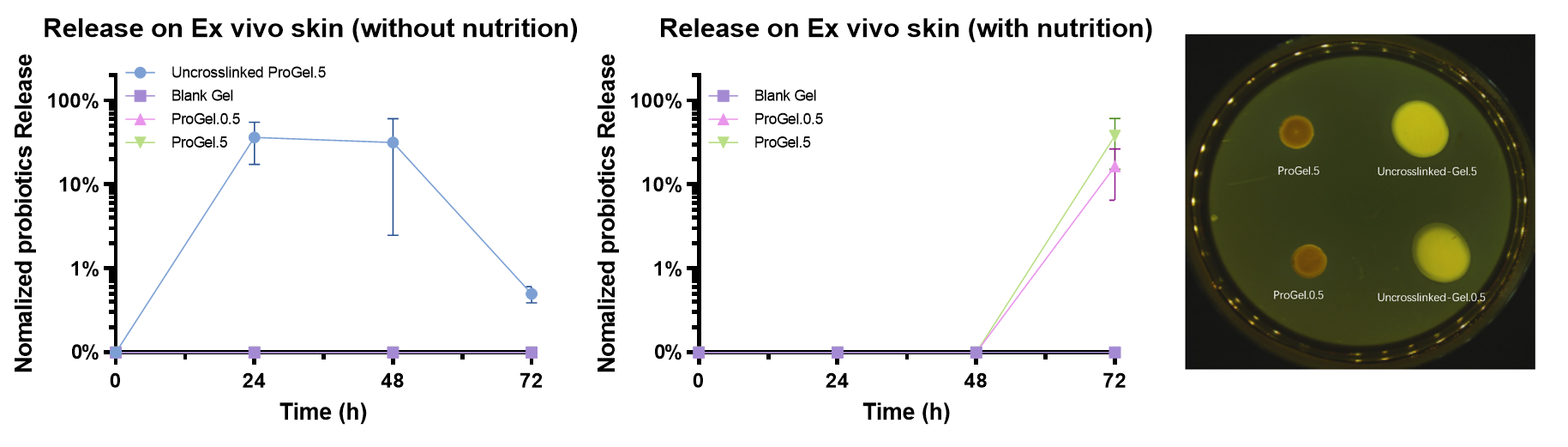


**Figure S6.** Probiotic Release Profiles from ProGels on Ex Vivo Skin Models with and without Nutritional Support. The left graph shows the release of probiotics from ProGels applied to ex vivo skin samples moistened with PBS. Probiotics entrapped in uncrosslinked gelatin were released within the first 24 hours, followed by a gradual decrease in titer. No release was detected in the groups with crosslinked Gelatin-ADH. The right graph depicts the release in a nutrition-rich environment (DMEM +10% FCS), where no release was observed in the first 48 hours, but a significant increase in probiotic titer occurred at 72 hours due to proliferation. The MRS agar plate (right) demonstrates the intactness of crosslinked Gelatin-ADH, confirming its ability to restrict probiotic release.

# Mesh size determination

The degree of crosslinking was determined using the Flory–Rehner equation, tailored for crosslinked networks formed in a diluent [3]. This determination was based on experimental swelling ratios derived from data recorded at 25°C

Volume fraction of dry gelatin ($v$*_s_*) in the swollen sample was specimen was deduced using Equation 3. For this evaluation, a dry weight of 0.15g crosslinked gelatin was observed to swell up to 1.39g when immersed in water. *ρ_g_* and *ρH_2_O* are the densities of the dried gelatin and water, taken as 1.35 g·cm^−3^ and 1 g·cm^−3^, respectively. Analogously, *v*^0^_s_, the volume ratio of the gels with respect to the dried gelatin immediately after gelation, was calculated.

$$\begin{aligned} v_{s}=\frac{m_{d}\rho_{H_{2}O}}{m_{d}\left( \rho_{H_{2}O}-\rho_{g} \right)+m_{w}\rho_{g}}\#\left( 3 \right) \end{aligned}$$

*M_c_*, the number average molecular weight between crosslinks, was then calculated based on Equation 4 with *M* taken as 87 500 g·mol^−1^, χ, the polymer–solvent interaction parameter, as 0.497, and$V_{1}$, the molar volume of water, as 18 ml·mol^−1^.

$$\begin{aligned} \frac{1}{M_{c}}=\frac{2}{M}-\frac{\left( \ln\left( 1-v_{s} \right)+v_{s}+\chi v_{s}^{2} \right)}{V_{1}\rho_{g}\left( \left( v_{s}^{0} \right)^{\frac{2}{3}}v_{s}^{\frac{1}{3}}-\frac{v_{s}}{2} \right)}\#\left( 4 \right) \end{aligned}$$

The mesh sizes, ξ, for equilibrium swollen gels is calculated as Equation 5, where M_r_ is the monomer mass of the repeating unit, taken as 100 g mol^−1^. *α* is an expansion factor relating this unperturbed value to the root-mean-square end-to-end distance, taken as 2.

$$\begin{aligned} \xi=2\alpha\left( \frac{M_{c}}{M_{r}} \right)^{\frac{1}{2}}\left( 2.21\text{Å} \right)v_{s}^{-\frac{1}{3}}\#\left( 5 \right) \end{aligned}$$

1. Cimen, Z., et al., *Injectable and Self-Healable pH-Responsive Gelatin-PEG/Laponite Hybrid Hydrogels as Long-Acting Implants for Local Cancer Treatment.* Acs Applied Polymer Materials, 2021. **3**(7): p. 3504-3518.

2. Sadeghi-Abandansari, H., et al., *Local co-delivery of 5-fluorouracil and curcumin using Schiff's base cross-linked injectable hydrogels for colorectal cancer combination therapy.* European Polymer Journal, 2021. **157**.

3. Wisotzki, E.I., et al., *Tailoring the material properties of gelatin hydrogels by high energy electron irradiation.* Journal of Materials Chemistry B, 2014. **2**(27): p. 4297-4309.
